# Economic Cost and Burden of Dengue in the Philippines

**DOI:** 10.4269/ajtmh.14-0139

**Published:** 2015-02-04

**Authors:** Frances E. Edillo, Yara A. Halasa, Francisco M. Largo, Jonathan Neil V. Erasmo, Naomi B. Amoin, Maria Theresa P. Alera, In-Kyu Yoon, Arturo C. Alcantara, Donald S. Shepard

**Affiliations:** Biology Department, University of San Carlos, Cebu City, Philippines; Brandeis University, Waltham, Massachusetts; Economics Department, University of San Carlos, Cebu City, Philippines; Department of Health Region 7, Cebu City, Philippines; Philippine Armed Forces Research Institute of Medical Sciences Virology Research Unit, Cebu City, Philippines; Department of Virology, Armed Forces Research Institute of Medical Sciences, Bangkok, Thailand; Philippine Health Insurance Corporation, Manila, Philippines

## Abstract

Dengue, the world's most important mosquito-borne viral disease, is endemic in the Philippines. During 2008–2012, the country's Department of Health reported an annual average of 117,065 dengue cases, placing the country fourth in dengue burden in southeast Asia. This study estimates the country's annual number of dengue episodes and their economic cost. Our comparison of cases between active and passive surveillance in Punta Princesa, Cebu City yielded an expansion factor of 7.2, close to the predicted value (7.0) based on the country's health system. We estimated an annual average of 842,867 clinically diagnosed dengue cases, with direct medical costs (in 2012 US dollars) of $345 million ($3.26 per capita). This is 54% higher than an earlier estimate without Philippines-specific costs. Ambulatory settings treated 35% of cases (representing 10% of direct costs), whereas inpatient hospitals served 65% of cases (representing 90% of direct costs). The economic burden of dengue in the Philippines is substantial.

## Introduction

Dengue illness is the most important mosquito-borne viral disease in the world.^1^ It is epidemic in the Philippines and considered one of its eight pervasive infectious diseases.^2^ From 2008 to 2012, the Philippines' Department of Health (DOH) reported 585,324 dengue cases, with a case fatality rate (CFR) of 0.55% or 3,195 deaths.^3^ Among the 10 Association of Southeast Asian Nations (ASEAN), the Philippines ranks fourth in the number of dengue cases.^4^–^7^ Four dengue virus (DENV; *Flavivirus*) serotypes are circulating in the Philippines^8^,^9^ transmitted by *Aedes aegypti* L. and *Ae. albopictus* Skuse (Diptera: Culicidae).^10^ Environmental risk factors and inconsistent preventive practices,^11^ in addition to urbanization, increasing population, inadequate public health infrastructure, poor solid waste management, and lack of an effective mosquito surveillance system contribute to the growing dengue challenge.^12^,^13^

Dengue surveillance in the Philippines depends mainly on disease reporting units (DRUs), which include sentinel health centers in the smallest government administrative unit (barangay), Rural Health Units (RHUs), municipal or city health offices, local hospitals, private clinics, and human quarantine stations under the Local Government Unit (LGU) surveillance system.^13^,^14^ The current surveillance system focuses on hospitalized cases, especially cases with severe dengue manifestation. Between 2008 and 2012, 96% of all reported dengue cases came from hospitalized settings, 57% of cases were reported by the public sector, and on average, 55% of reported cases were diagnosed with dengue hemorrhagic fever (DHF) or dengue shock syndrome (DSS).^3^ The new dengue classification set by the World Health Organization (2009)^15^ was endorsed in the Revised Dengue Clinical Case Management Guidelines by Dr. Enrique T. Ona, Philippine DOH Secretary, in March of 2012.^16^ However, the Philippines DOH surveillance system has continued to use the traditional dengue classification system, because some hospitals have not yet adopted the new classification scheme.^14^ Admission data from the Philippine Health Insurance Corporation (PhilHealth), the country's national health insurance program, indicated that, on average, 72% of PhilHealth members who were hospitalized for dengue were treated in the private sector between 2010 and 2012.^17^ Using definitions from the Philippines Integrated Disease Surveillance and Response (PIDSR),^14^ during this period, PhilHealth classified dengue cases into two levels of severity, dengue without major complications (level 1) and dengue with major complications (level 2),^17^ using the traditional dengue classification.

Passive surveillance systems capture only a proportion of dengue cases in endemic countries, making underreporting of dengue cases a chronic challenge in endemic countries.^5^,^12^,^18^–^22^ Many Asian countries rely on passive dengue surveillance systems, which are based mainly on clinical diagnosis without laboratory confirmation.^23^–^26^ A recent study of dengue burden in southeast Asia estimated that only 13% of all dengue cases in that region are captured and reported by the surveillance systems.^5^ Based on a regression model, that study estimated a reporting rate of 14.3% for the Philippines or an expansion factor of 7.0 (i.e., for each reported case, there are 7.0 clinically apparent dengue cases).^5^

Borja^11^ estimated the economic and human burden of dengue in the Philippines in 2007 and reported the annual burden of dengue at 18,074 disability adjusted life years (DALYs), with an average case cost of $210.75 (adjusted to 2012 US dollars), which covers the cost of diagnosis, treatment, and income loss for patients and caregivers. Using the original classification of dengue severity, her study estimated that the cost per case of DHF or DSS was nearly three times the cost of dengue fever (DF) and that the cost of treatment in the private sector was double that in the public sector.^11^ Although useful, that study focused only on hospital settings and did not adjust for underreporting of dengue cases under the DOH surveillance system.

In addition to dengue's burden on a household, the illness can adversely impact a country's economy through loss of productivity caused by illness and pre-mature death, increased healthcare costs, and possible reduction in tourism.^5^ Estimating the actual economic cost and disease burden of dengue illness in the Philippines is relevant to inform policymakers, set priorities in health policy, and implement disease control strategies. This paper aims to estimate (1) the country's annual number of dengue episodes by adjusting the reported national dengue surveillance for underreporting using an adjustment factor based on an empirical case study implemented in Punta Princesa, Cebu City, Philippines;^27^ (2) the average direct medical cost of a dengue episode by setting (ambulatory and hospitalized) and sector (public and private) using the macro-costing method; and (3) the aggregate annual direct medical cost of dengue in the country by sector and setting.

## Methods

This study consists of three parts. The first part estimates the number of dengue episodes, the second part derives the cost of dengue treatment per case by setting and sector, and the third part computes the aggregate direct medical cost of dengue based on the prior two components.

### Part 1: Disease burden of dengue.

#### National and state surveillance systems.

We performed a systematic review of the Philippines national and regional dengue surveillance systems to understand their processes and defined their strengths and weaknesses. We obtained dengue incidence by year, setting, sector, and severity from the Philippines' national and regional dengue surveillance systems. We collected reported national dengue cases by setting (ambulatory and hospitalized) and sector (public and private) as well as deaths from the DOH National Epidemiology Center (NEC; Manila, Philippines) for the years 2008–2012.^3^ The NEC uses the traditional dengue case classification as described in the PIDSR system in accordance with international health regulations of the World Health Organization in 2005.^14^

The Philippines' dengue surveillance system involves reporting across the spectrum of dengue illnesses (DF, DHF, and DSS) from health centers in the smallest administrative unit (barangay) to city hospitals, to district or provincial hospitals to regional hospitals, and finally to the NEC by coordinators and officers trained by PIDSR.^14^ The Epidemiological Case Investigation form (Supplemental Figure 1) is usually filled in by public health nurses (PHNs), medical technicians, or rural health midwives (RHMs).

**Figure 1.**
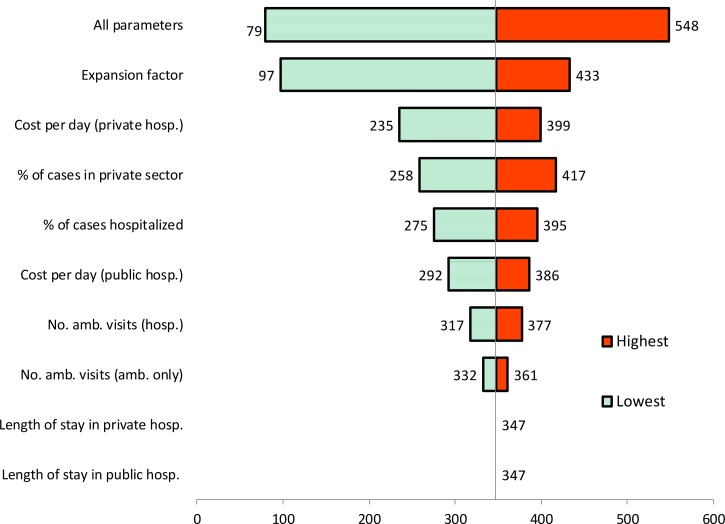
Variability of total dengue costs according to parameters included in the sensitivity analysis (2012 US millions of dollars). amb. = ambulatory; hosp. = hospital; no. = number.

Using the standardized clinical case definitions, the data are reported from the barangay, municipal levels, and other data-reporting units on the Case Report Form submitted to the DOH–NEC (Supplemental Figure 2). For hospitalized patients, the case classification is based on the hospital discharge diagnosis by the attending physician. For ambulatory cases, diagnosis is made by a medical doctor. Standard case definition of dengue cases follows the traditional dengue classification.^14^ Briefly, a suspected DF is an acute febrile illness that lasts for 2–7 days with two or more of the following signs: headache, retro-orbital pain, myalgia, arthralgia, rash, hemorrhagic manifestations, and leucopenia. A probable DF is a suspected case with one or more supportive laboratory tests, whereas a confirmed case is a suspected case that is laboratory-confirmed. DHF is a probable or confirmed case of dengue with hemorrhagic tendencies^28^ and other indications. DSS is DHF plus evidence of circulatory failure and altered mental status.^14^ Laboratory testing is rarely done because it requires that the patient bears the cost of the test.

Because of the variability in the number of dengue cases among years, we computed the average annual number of dengue cases for the years 2008–2012 to generate a stable estimate of the total projected annual number of dengue cases. To project the average annual number of dengue cases, we addressed incomplete data by allocating the unknown cases by setting and sector based on the percentage of each category to the total number of cases with known categories.

#### Adjustment factor: Punta Princesa case study.

As part of a study conducted by the Philippine Armed Forces Research Institute of Medical Sciences (AFRIMS) Virology Research Unit (PAVRU), a prospective cohort study in Punta Princesa was conducted between March of 2012 and March of 2013.^27^ This cohort study was approved by the Institutional Review Boards of Vicente Sotto Memorial Medical Center, Cebu City, Philippines and Walter Reed Army Institute of Research (WRAIR). Written informed consent was obtained from all study respondents or their parents, if applicable, and oral assent was obtained from children older than 12 years of age. Active surveillance using weekly short messaging services (SMSs), phone calls, or home visits was undertaken to check the health status of the cohort of 1,000 participants. For individuals with fever history within the previous 7 days, acute blood samples were drawn and tested for serotype-specific DENV using hemi-nested reverse transcription polymerase chain reaction (PCR),^29^ and acute and 14-day convalescent blood samples were collected and tested using immunoglobulin M (IgM)/IgG enzyme immunoassay (EIA). Symptomatic dengue cases, captured by the active surveillance system and confirmed by laboratory testing, were compared with reported dengue cases captured by the official surveillance system of the Cebu City Health Department.

#### Delphi panel and expert opinion.

Thirty-four national and international experts participated in a 1-day Delphi panel workshop in Cebu City, Philippines in 2013 to address gaps in knowledge related to some aspects of dengue treatment in the country. These experts provided insights from academic, public, and private perspectives. They estimated parameters, such as the percentage of dengue cases treated in a hospitalized setting, the percentage of hospitalized dengue cases treated in private and public hospitals, and an overall adjustment factor for dengue cases in the Philippines.

We used a Tukey outlier test to identify possible outliers and ensure that our central value estimates were unbiased. We set inner fences at a distance of 1.5 times the interquartile range (IQR) below the first quintile and above the third quintile. We located outer fences at a distance of 3 IQR below the first quintile and above the third quintile.^30^,^31^

### Part 2: Direct medical cost of dengue episodes.

We used the macrocosting methodology^32^ and medical bills of hospitalized dengue cases to estimate the average cost of a bed day in public and private hospitals. We collected original cost data from two private and two public hospitals; PhilHealth identified these four hospitals as hospitals with the highest numbers of reported dengue cases for Manila and Cebu in 2011. We valued a hospital outpatient visit as 32% of a bed day and a non-hospital ambulatory visit at 80% of the cost of an outpatient hospital visit based on international experience.^32^ We combined this information with data from Tsilaajav^33^ to obtain unit costs from 10 hospitals (3 private and 7 public hospitals).

We calculated the average length of stay (LoS) of a hospitalized case by sector from PhilHealth admission data for the years 2010–2012 that were weighted to the overall PhilHealth membership. To obtain the cost of hospitalization by sector, we multiplied the average LoS for dengue patients in that sector by the cost per bed day equivalent. A hospitalized case uses ambulatory services in addition to the hospital stay. Based on international experience, we assumed that a dengue patient would have, on average, 4.6 ambulatory visits, of which 1.7 ambulatory visits would take place in a hospital setting and 2.9 ambulatory visits would take place in other ambulatory settings.^22^

To compute the cost of cases receiving only ambulatory care, we assumed that these patients would have, on average, 4.2 visits based on international experience.^22^ On average, 1.3 of these visits would be in a hospital setting, and 2.9 of these visits would be in other settings.

### Part 3: Aggregate direct medical cost of dengue.

#### Use of adjustment factor.

To project the average annual number of clinically diagnosed dengue cases in the Philippines between 2008 and 2012, we adjusted the reported number of clinically diagnosed dengue cases using the adjustment factor derived from the Punta Princesa case study.

We allocated cases to settings and sectors based on Delphi panel experts' opinion. To compute the direct medical cost of dengue illness in the Philippines, we multiplied the projected dengue cases times the cost per case by sector and setting.

#### Sensitivity analyses.

To address the inherent uncertainty associated with the parameters used to estimate the average annual aggregate direct medical cost of dengue, we used probabilistic sensitivity analyses for individual parameters as well as all of the parameters combined based on the valid values. We used the following variables: overall expansion factor (range = 2–10), proportion of cases hospitalized (range = 40–80%), proportion of cases treated in the private setting (range = 30–65%), and cost per bed day in public (range = $29.67–$94.42) and private hospitals (range = $52.98–$179.64). The lower values for cost per bed days at public and private hospitals were obtained by multiplying the average cost per bed day by a ratio of the average charge per case treated in small hospitals to the average charge per case treated in specialized hospitals, the number of ambulatory visits for hospitalized cases (non-sample error confidence interval [CI] = 2.3–6.9), the number of ambulatory visits for ambulatory cases (non-sample error CI = 2.1–6.3), and the average LoS in both public (CI = 4.36–4.38) and private (CI = 4.02–4.04) hospitals. Results are presented as mean values with 95% CIs. For each sensitivity analysis, we performed 1,000 Monte Carlo simulations with an associated triangular distribution. Results are presented as mean values and 95% CIs.

## Results

### Part 1: Disease burden of dengue.

#### Reported dengue cases.

Adjusting for the unknown category, we calculated that the Philippines had an annual average of 117,065 reported dengue cases between 2008 and 2012. Of these cases, 57% (*N* = 67,008) were from public sources, and 95% (111,259) were from hospital settings. On average, 53% (*N* = 62,380) of the reported cases were diagnosed as DHF or DSS, and 47% were diagnosed as DF. Table 1 displays numbers of reported dengue cases by disease classification, sector, and setting for the years 2008–2012. The Research Institute for Tropical Medicine (RITM) has determined the serotype for 1,294 dengue cases from 2008 to 2012. The distribution was 20% for DENV-1, 18% for DENV-2, 54% for DENV-3, and 8% for DENV-4.^34^ In a prospective community-based study of infants from 2007 to 2009, DENV-3 also predominated.^35^

#### Adjustment factor: Punta Princesa case study.

Partial results for the hemi-nested PCR of blood samples of volunteers in Punta Princesa from March to October of 2012 and the corresponding incidence rate of dengue from the passive surveillance system of the Cebu City Health Department showed an average reporting rate of 13.28% of the total symptomatic dengue cases. Thus, the underreporting rate was 86.72%, and the adjustment factor was 7.2, meaning that, for each reported case, there are 7.2 actual dengue cases.

#### Delphi panel and expert opinion.

Of 34 experts who participated in the Delphi panel, 23 experts were free of possible commercial conflicts and eligible to provide their expert opinion. Based on Tukey test results, the highest value of 15 for the overall expansion factor was considered a possible outlier, and therefore, we chose the next value of 10 as the highest valid value. For the proportion of cases treated in private setting, the value of 70% was considered a possible outlier, and therefore, we selected the next value of 65% as the highest valid value. On average ± standard deviation (SD), the panel estimated that 65% (±12%) of all dengue patients are treated in a hospitalized setting. The expert panel suggested that 46% (±11%) of hospitalized dengue cases are treated in private settings. As for the overall expansion factor, the Delphi panel estimated an average of 5.9 (±3.5).

### Part 2: Direct medical cost of a dengue episode.

As discussed further below, the total direct medical cost of a treated hospitalized case averaged $772.46 in private hospitals and $387.84 in public hospitals. The average cost of a dengue case treated only in an ambulatory setting was $79.43 in the public sector and $168.31 in the private sector.

### Part 3: Aggregate direct medical cost of dengue.

Adjusting the average annual reported dengue cases between 2008 and 2012 (117,065) by the adjustment factor from the Punta Princesa case study (7.2) yielded a total number of 842,867 cases per year. As presented in Table 2, ambulatory cases had a higher adjustment factor (51.0) than hospitalized cases, suggesting that only 2% of all ambulatory cases are captured and reported by the national surveillance system. Ambulatory cases treated in the private sector had the highest adjustment factor of 110.2 and the lowest reporting rate of 1% followed by ambulatory cases treated in the public sector (an adjustment factor of 35.0 and a reporting rate of 3%). Cases reported from public hospitals had the lowest adjustment factor of 4.7 and the highest reporting rate of 21% followed by hospitalized cases in the private sector (an adjustment factor of 5.2 and a reporting rate of 19%).

Table 3 presents the cost per case and aggregate direct medical cost of dengue in the Philippines for 2008–2012. The total annual aggregate cost was US $345 million. Of this cost, 89.7% was for hospitalized cases and 10.3% was for ambulatory cases. The share of the private sector was 63.1% compared with 36.9% in the public sector.

### Sensitivity analysis.

The sensitivity analysis shows that the expansion factor creates the highest uncertainty on the aggregate medical cost of dengue. Under the best estimate for the expansion factor (7.2), the estimated total cost was US $345 million. However, if the expansion factor were at the minimum or maximum valid values, then the aggregate cost would decrease by 72% or increase by 25%. The uncertainty associated with the cost per hospitalized day in the private sector generated the second largest variation: −32% and +15%. The proportion of dengue patients treated in the private setting generated a variation in the total cost ranging from −26% to +20%. The proportion of dengue cases hospitalized generated the fourth variation: −21% and +14%. The impact of patients' lengths of stay in both public and private hospitals had a very small range (under 0.01%). Simulating our parameters together would increase the cost by 58% or decrease it by 77%. The results are shown in Figure 1.

## Discussion

To understand the economic cost and burden of dengue in the Philippines, we used existing data and expert opinion to estimate the number of dengue cases and aggregate direct medical costs in the Philippines for 2008–2012. The analysis of the national surveillance system showed, as expected, the ability of the system to capture severe cases: more than one-half of the reported cases (53%) were severe dengue cases, which were treated mainly in hospitalized settings. However, a prospective study in Manila, the Philippines concluded that only one-third of dengue patients suffered from DHF,^36^ and a dengue cost model suggested that between 1.2% and 6% of all symptomatic dengue cases manifest in the disease's severest form.^37^ These estimates suggest that less severe manifestations of the disease are substantially underreported in the Philippines.

To adjust for underreporting, we compared the results of the cohort study in Punta Princesa, Cebu City with modeled estimates generated for southeast Asian countries, including the Philippines,^5^ which estimate that 14.3% of the total symptomatic dengue episodes were reported in the Philippines with an overall adjustment factor of 7.0. Moreover, Borja^11^ noted that, in the cities of Manila, Muntinlupa, Baguio, Iloilo, Cebu, and Davao, only 19% of dengue cases were reported, giving an adjustment factor of 5.3. For this study, we used the most recent adjustment factor (i.e., 7.2 from Punta Princesa, Cebu City). Our result shows that dengue is a major health problem in the Philippines, with 842,867 estimated annual cases of symptomatic dengue (based on the adjusted cases for the years 2008–2012), of which over 546,659 were hospitalized cases.

The weighted average medical cost of a hospitalized case was $565.04 (CI = $351.88–$673.18) and $120.38 (CI = $61.18–$157.49) for an ambulatory case. The cost per visit in a private setting was nearly two times the cost of treatment in a public setting. Our estimated costs per case are more than two times the previous estimates for the Philippines ($207.40 for a hospitalized case and $55.29 for an ambulatory case; adjusted for inflation).^5^ A prior estimate for the Philippines extrapolated the cost per case from a relatively small number of costing studies in other southeast Asian countries based on the correlation between gross domestic product (GDP) per capita and cost per case. Our present estimate considered the specific characteristics of the Philippines healthcare system, such as its relatively high insurance coverage. Additionally, the prior estimates of cost per dengue case used in the regression analysis were based on data generated mainly from the public sector. In contrast, our estimates were derived from data collected and analyzed from both public and private settings.

The direct medical cost associated with dengue is estimated at $345 million or $3.26 per capita. This is 54% higher than an earlier estimate for 2010 of $223.8 million extrapolated from neighboring countries without Philippines-specific costs.^5^ The weighted average cost of treatment per case was $409, representing 16% of the Philippines' per capita GDP ($2,587 in 2012).^38^ To obtain the total cost of a non-fatal dengue case, we obtained the indirect cost by adjusting results of a previous study^5^ for inflation using the US GDP deflator.^39^ We derived the direct non-medical cost based on the ratio of this component to indirect cost in a multi-country study for hospitalized (0.4214) and ambulatory (0.0699) cases.^22^ The resulting cost of an ambulatory case ($138.80) was a higher share (7%) of per capita GDP than that in other countries with comparable data, such as Panama ($352 or 4% of per capita GDP [$9,534 in 2012])^18^ and Puerto Rico ($2,654 or 10% of per capita GDP [$27,678 in 2012]).^19^ Similarly, the cost of a hospitalized case in the Philippines ($617.37 or 30% of per capita GDP) was a higher share of GDP per capita than Panama ($1,129 or 12% of per capita GDP) but not quite as high as in Puerto Rico ($11,451 or 41% of per capita GDP). These comparisons indicate that dengue has a relatively large impact on both households (which incur most of the private sector direct medical, direct non-medical, and indirect costs) and the public sector (which supports much of the public sector direct medical costs) in the Philippines.

This study has several limitations. First, we depended on expert opinion to allocate cases among healthcare settings. Because many experts worked in hospitals, they may have overestimated the share in hospital settings. In this case, our computation would have overestimated the aggregate direct medical cost, which is illustrated in the sensitivity analysis. Second, we assumed that the proportion of ambulatory dengue cases treated in the private sector is the same as that proportion of hospitalized patients treated in the private sector. If a lower share of ambulatory cases was treated in the public sector, then we underestimated the aggregate cost, which is highlighted in the sensitivity analysis. Third, our costs were based on data from tertiary hospitals; although we have endeavored to adjust for lower costs in other hospitals, our costs might have been overestimated, which is highlighted in the sensitivity analysis. Fourth, our analysis focused on the direct medical cost of dengue in the Philippines, with only a preliminary analysis of indirect costs. Fifth, because we lacked data from the Philippines, we estimated numbers of visits per episode from international data. Sixth, the macrocosting approach used in this study provides an overall cost per hospitalization. This approach does not provide a breakdown of hospitalized cases by illness severity. Our prior work has found that the treatment setting (hospitalized versus ambulatory) was the primary determinant of the cost of a dengue episode.^19^,^25^ Detailed person-level costing would be needed for additional analyses of dengue costs by severity. To our knowledge, such cost data are not currently available for the Philippines. Seventh, the aggregate overall cost of dengue should include the cost of disease surveillance and vector control, but costs of these activities were not available.

Better understanding of the disease burden and cost of dengue in the Philippines is needed to prioritize strategies to control dengue, including new technologies to control the vector and virus. Although the current surveillance system has several strengths, it does not capture most of the dengue cases treated in an ambulatory setting in the private sector. Adjusting for underreporting shows that the cost of dengue illness, excluding the surveillance and control measures, represents 0.15% (i.e., $0.627 billion per $425.2 billion) of the total GDP in the Philippines for 2012. Based on the GDP per capita, the cost of this illness is equivalent to the aggregate annual output of 242,000 people.^38^ These costs highlight the need to evaluate current and potential dengue control strategies in the Philippines.

## Supplementary Material

Supplemental Figures.

## Figures and Tables

**Table 1:** Numbers of dengue cases reported by sector, setting, disease classification, and year (2008–2012)

	2008	2009	2010	2011	2012	Average	Percentage
Breakdown by sector and setting							
Ambulatory public	211	997	8,523	6,934	6,173	4,567	4
Ambulatory private	44	22	1,490	1,513	2,824	1,239	1
Hospitalized public	32,031	29,358	91,144	69,684	89,987	62,441	53
Hospitalized private	13,951	24,265	69,981	47,845	88,048	48,818	42
Breakdown by classification							
DF	16,831	23,938	88,509	66,485	77,660	54,684	47
DHF and DSS	29,407	31,004	82,629	59,490	109,371	62,380	53
Total	46,238	54,942	171,138	125,975	187,031	117,065	100

**Table 2:** Reported and adjusted numbers of clinically diagnosed dengue cases by setting and sector (annual average; 2008–2012)

Item and setting	Public	Private	Total	Percentage
Reported clinically diagnosed dengue cases				
Ambulatory	4,567	1,239	5,806	5
Hospitalized	62,441	48,818	111,259	95
Total	67,008	50,057	117,065	100
Percentage	57	43	100	
Adjusted clinically diagnosed dengue cases				
Ambulatory	159,740	136,467	296,207	35
Hospitalized	294,805	251,854	546,659	65
Total	454,546	388,321	842,867	100
Percentage	54	46	100	
Adjustment factor				
Ambulatory	35.0	110.2	51.0	
Hospitalized	4.7	5.2	4.9	
Total	6.8	7.8	7.2	

The allocation between settings and sectors was based on the Delphi panelists' expert opinion.

**Table 3:** Average annual direct medical cost of dengue cases by setting and sector 2008–2012 (in 2012 US dollars)

Setting	Public	Private	Combined
Ambulatory cases			
Hospital outpatient departments			
Cost per visit	$21.94	$46.50	$33.25
Number of visits	1.3	1.3	1.3
Cost per case in outpatient department	$28.52	$60.44	$43.23
Other ambulatory settings			
Cost per visit	$17.55	$37.20	$26.60
Number of visits	2.9	2.9	2.9
Cost per case in other settings	$50.90	$107.87	$77.15
Total cost per ambulatory case	$79.43	$168.31	$120.38
Number of adjusted ambulatory cases	159,741	136,467	296,208
Aggregate cost of ambulatory cases	$12,688,000	$22,969,000	$35,657,000
Percentage of aggregate medical cost	3.7	6.7	10.3
Hospitalized cases			
Hospitalized episode			
Cost per bed day	$68.57	$145.30	$102.46
LoS	4.37	4.03	4.21
Cost per hospitalization	$299.64	$585.55	$431.36
Ambulatory care for hospitalized cases			
Hospital setting			
Cost per visit	$21.94	$46.50	$33.25
Number of visits	1.7	1.7	1.7
Cost per case	$37.30	$79.04	$56.53
Other ambulatory setting			
Cost per visit	$17.55	$37.20	$26.60
Number of visits	2.9	2.9	2.9
Cost per case	$50.90	$107.87	$77.15
Cost of ambulatory care per hospitalized case	$88.20	$186.91	$133.68
Total cost per hospitalized case	$387.84	$772.46	$565.04
Adjusted number of hospitalized cases	294,805	251,854	546,659
Aggregate cost of hospitalized cases	$114,337,000	$194,548,000	$308,885,000
Percentage of aggregate medical cost	33.2	56.5	89.7
Aggregate direct medical cost	$127,025,000	$217,517,000	$344,542,000
Percentage of aggregate medical cost	36.9	63.1	100.0
