# Supplementary material for: Economic Cost and Burden of Dengue in the Philippines
Source: Am J Trop Med Hyg. 2015 Feb 4;92(2):360–6. doi: 10.4269/ajtmh.14-0139 (PMC4347342; doi:10.4269/ajtmh.14-0139)
Supplement: Supplementary file 1 [file SD5.pdf]

(FORM 6 DENGUE Page 1)

Republic of the Philippines
CITY HEALTH DEPARTMENT
City of Cebu

EPIDEMIOLOGICAL CASE INVESTIGATION

Date of Investigation ______

Investigator (Name / Position) ______

Informant / Relation ______

I. PERSONAL DATA:

Name ______ Age ______ Sex ______ C.S. ______

Complete Address ______

Length of Stay in above Address ______

Provincial / Previous Address ______

Occupation ______

School / Office Address ______

Father's Name ______ Mother's Name ______

II. CLINICAL DATA:

Date of Onset ______ Duration (days) ______

Outcome : ( ) Still Sick ( ) Recovered ( ) Died

Consulted : (Y) (N) If yes, when ______ where ______

Admitted : (Y) (N) If yes, when ______ where ______

Treatment: ( ) Antipyretics ( ) IV Fluids ( ) Blood Transfusion

Others ______

Signs & Symptoms: ( ) Fever ( ) Chills ( ) Headache ( ) Abdo. Pain

( ) Epistaxis ( ) Cough Others ______

Complication (s): (Y) (N) What ______

Laboratory Exams:	*When*	*Where*	*Result*
CBC			
Platelet Det.			
Others			

Diagnosis: Tentative ______ Final ______

(FORM 6 DENGUE Page 2)

III. EPIDEMIOLOGICAL NOTES:

Do you use protection against mosquitoes (Y) (N)

If yes, what ______

Presence of mosquito breeding places:

Stagnant water: ( ) Dranaige canals ( ) Excavation ( ) Old tires
( ) Empty containers ( ) Others ______

Shaded areas under trees

Knowledge of other Dengue cases in the area:

______

______

IV. OTHER PERTINENT DATA:

______

______

______

______

V. INTERVENTIONS DONE:

______

______

______

______

SUBMITTED BY:

______ (Printed name, Position & Signature)

______ Medical Officer

Date submitted: ______

Received by:

______ Date Received ______

Supplemental Figure 1. Epidemiological case investigation.

Philippine Integrated Disease Surveillance and Response

Case Report Form

Dengue (ICD 10 Code: A90-A91)

Region: ____________________________ **Province**: ____________________________ **Municipality/City**: ____

Name of DRU: ____ **Type**: ☐RHU ☐CHO ☐Gov't Hospital ☐Private Hospital ☐Clinic

Address: ____ ☐Private Laboratory ☐Public Laboratory ☐Seaport/Airport

Patient No.	Patient's Full Name	Age	Sex (F/M)	Date of Birth	Complete Address	Admit-ted?	Date admit-ted/seen/ consulted	Date onset of illness	Type	Case classifi-cation	Out-come
				___/___/___			___/___/___	___/___/___			
				___/___/___			___/___/___	___/___/___			
				___/___/___			___/___/___	___/___/___			
				___/___/___			___/___/___	___/___/___			
				___/___/___			___/___/___	___/___/___			
Response Codes / Instructions	Indicate First name, Middle name, Last name	*Age*: Indicate D - days M - months Yr. - years *Sex*:F - Female M - Male		mm/dd/yy	Specify Street/Purok/Subdivision, House #, Barangay, Municipality/City, Province	Y - Yes N- No	mm/dd/yy	mm/dd/yy	W—with warning signs N– no warn-ing signs S-Severe Dengue	S - Sus-pect P - Prob-able C - Con-firmed	A - Alive D - Died (specify date) U - Un-known

Clinical Case Definition/Classification:

Dengue without Warning signs.

- ***Suspect***

A previously well person with acute febrile illness of 2-7 days duration plus two of the following:

Headache, Body malaise, Myalgia, Arthralgia, Retro-orbital pain, Anorexia, Nausea, Vomiting, Diarrhea, Flushed skin, Rash (petecheal, Herman's sign)

- ***Probable***

A suspect case **plus:**

Laboratory test, at least CBC (leucopenia with or without thrombocytopenia) and/or Dengue NS1, antigen test or dengue IgM antibody test (optional)

- ***Confirmed***:
  - Viral culture isolation,
  - Polymerase Chain Reaction

Dengue with Warning Signs

,A previously well person with acute febrile illness of 2-7 days duration plus any one of the following:

- Abdominal pain or tenderness
- Persistent vomiting
- Clinical signs of fluid accumulation
- Mucosal bleeding
- Lethargy, restlessness
- Liver enlargement
- Laboratory: increase in Hct and/or decreasing platelet count

Severe Dengue

A previously well person with acute febrile illness of 2-7 days duration and any of the clinical manifestations for dengue with or without warning signs,

Plus any of the following:

Severe plasma leakage leading to

- Shock
- Fluid accumulation with respiratory distress

Severe bleeding

Severe organ impairment

- Liver: AST or ALT $\geq$1000
- CNS: e.g. seizures, impaired consciousness
- Heart: e.g. myocarditis
- Kidneys: e.g. renal failure

Philippine Integrated Disease Surveillance and Response

Case Report Form

Dengue (ICD 10 Code: A90-A91)

Patient No.	Patient's Full Name	Age	Sex (F/M)	Date of Birth	Complete Address	Admit-ted?	Date admit-ted/seen/ consulted	Date onset of illness	Type	Case-Classifi cation	Out-come
				___/___/___			___/___/___	___/___/___			
				___/___/___			___/___/___	___/___/___			
				___/___/___			___/___/___	___/___/___			
				___/___/___			___/___/___	___/___/___			
				___/___/___			___/___/___	___/___/___			
				___/___/___			___/___/___	___/___/___			
				___/___/___			___/___/___	___/___/___			
				___/___/___			___/___/___	___/___/___			
				___/___/___			___/___/___	___/___/___			
				___/___/___			___/___/___	___/___/___			
				___/___/___			___/___/___	___/___/___			
Response Codes / Instructions	Indicate First name, Middle name, Last name	*Age*: Indicate D - days M - months Yr. - years *Sex*:F - Female M - Male		mm/dd/yy	Specify Street/Purok/Subdivision, House #, Barangay, Municipality/City, Province	Y - Yes N- No	mm/dd/yy	mm/dd/yy	W - with Warning signs N– no warn-ing signs S—Severe Dengue	S– Suspect P- Prob-able C – confirmed	A - Alive D - Died (specify date) U - Unknown

SUPPLEMENTAL FIGURE 2. Case report form dengue (ICD 10 Code: A90-A91).
